# Secondary Angle-Closure Glaucoma Associated With Congenital Acorea That Developed Into Endophthalmitis After Glaucoma Drainage Implant Surgery: A Case Report

**DOI:** 10.7759/cureus.92358

**Published:** 2025-09-15

**Authors:** Kenya Yuki, Yumi Matsuura, Mizuki Yaginuma, Takashi Negishi, Masayoshi Shinjoh

**Affiliations:** 1 Department of Ophthalmology, Nagoya University Graduate School of Medicine, Nagoya, JPN; 2 Department of Ophthalmology, Keio University School of Medicine, Tokyo, JPN; 3 Department of Pediatrics, Keio University School of Medicine, Tokyo, JPN; 4 Department of Ophthalmology, Juntendo University, Tokyo, JPN

**Keywords:** acorea, angle closure glaucoma, endophthalmitis, glaucoma drainage implant, pupilloplasty

## Abstract

We report a rare case of unilateral secondary angle-closure glaucoma associated with congenital acorea in a 10-month-old male infant. Acorea is a rare congenital eye abnormality characterized by the complete absence of the pupil. The left cornea of this patient measured 13.0 mm vertically and horizontally, with opacities observed. The anterior chamber was absent, and the pupil was completely occluded. Intraocular pressure (IOP) was 26 mmHg, and axial length was 25.3 mm. The fundus was not visible due to acorea. The patient was diagnosed with secondary angle-closure glaucoma due to acorea. To relieve angle closure, a pupil was created under general anesthesia. After blunt dissection with viscoelastic, a vitreous cutter was inserted through a corneal incision, and central iridectomy was performed. Anterior chamber depth was normalized immediately. Despite initial surgical pupil formation and two trabeculotomies, IOP remained uncontrolled, requiring sequential implantation of an Ahmed glaucoma valve and a Baerveldt glaucoma implant. Although the postoperative IOP stabilized, the patient developed endophthalmitis six months later due to *Haemophilus influenzae*. Despite intensive treatment, the infection developed into phthisis bulbi after tube exposure. This case highlights the potential for secondary angle-closure glaucoma in patients with acorea and emphasizes the risk of severe postoperative complications, including endophthalmitis, following glaucoma drainage device implantation in infants.

## Introduction

Acorea is a rare congenital ocular abnormality characterized by the complete absence of the pupil [1]. Patients with acorea may present with associated anomalies such as microphthalmia, congenital cataracts, iridocorneal dysgenesis, and secondary congenital glaucoma [1-4]. Bobrova et al. reported a case of secondary angle-closure glaucoma caused by congenital acorea in a one-month-old child, which was successfully controlled by peripheral iridectomy and dissection of iris-corneal synechiae. However, secondary glaucoma in these patients often requires surgical intervention when intraocular pressure (IOP) cannot be adequately controlled [4].

For refractory childhood glaucoma, glaucoma drainage implants such as the Ahmed glaucoma valve (AGV) and the Baerveldt glaucoma implant (BGI) are widely used to lower IOP. In a meta-analysis of 32 studies including 1,480 eyes, the mean IOP reduction was comparable between AGV and BGI, and postoperative complication rates were also similar (AGV: 28%; BGI: 27%) [5].

Herein, we report the first case of secondary angle-closure glaucoma associated with congenital acorea that progressed to endophthalmitis following glaucoma drainage implant surgery. This case highlights the unique clinical course and underscores the importance of recognizing potential complications in pediatric patients with rare congenital anomalies.

## Case presentation

We report the case of a 10-month-old boy who presented with corneal opacity in the left eye. At one month of age, during a routine infant checkup, this patient was noted to have an abnormality in the left iris. At 10 months of age, a primary care ophthalmologist observed elevated IOP in the left eye, and the patient was referred to a university hospital. At that time, IOP was 17 mmHg in the right eye and 40 mmHg in the left eye. Peter’s anomaly was suspected, and the patient was subsequently referred to Keio University Hospital (Tokyo, Japan). Based on these clinical findings, secondary angle-closure glaucoma of the left eye was suspected. Therefore, an examination was performed under general anesthesia for further evaluation.

**Figure 1:**
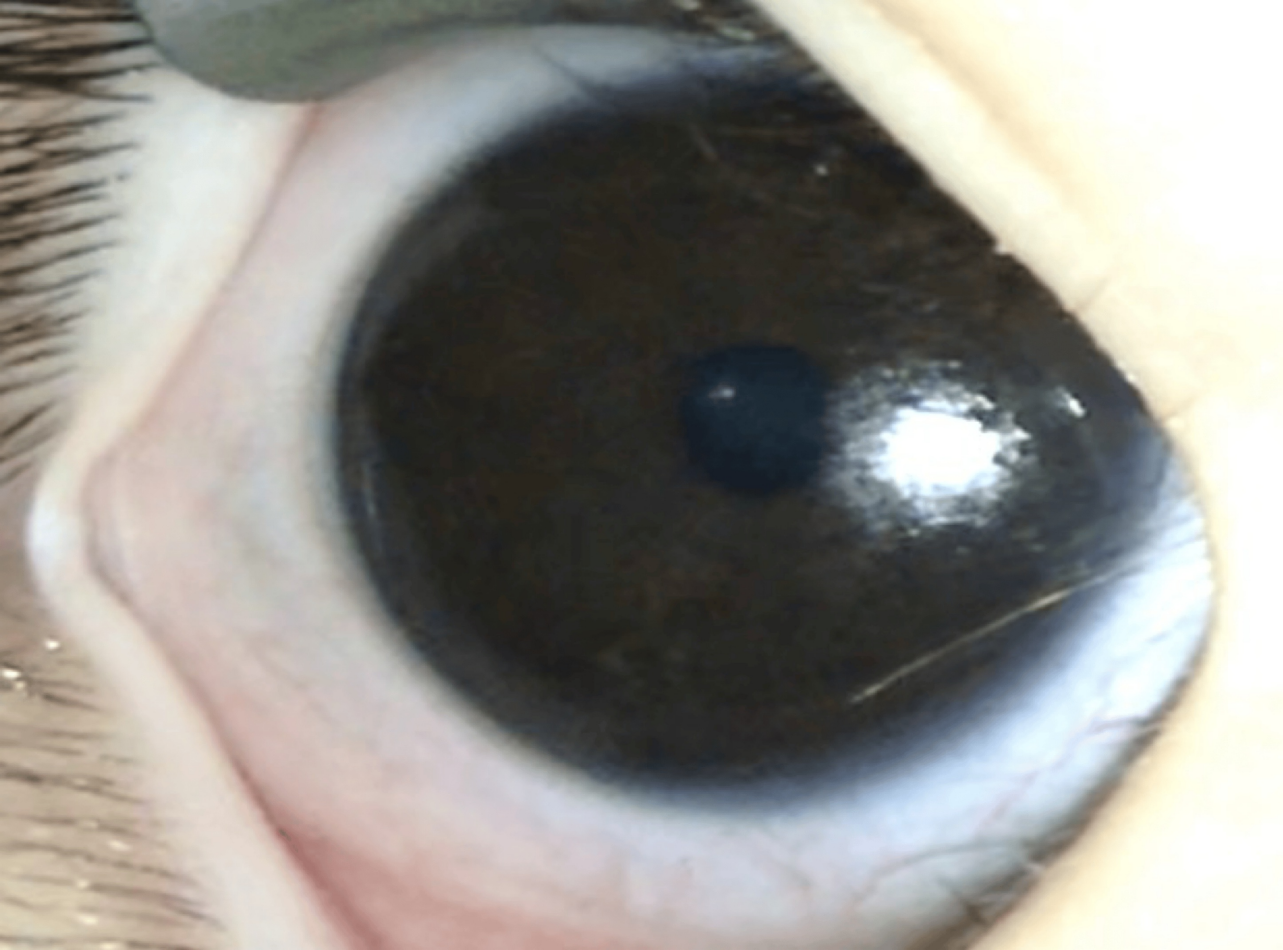
Anterior segment photograph of the right eye showing no significant abnormalities The iris and pupil exhibit no apparent morphological abnormalities

The patient was born at 41 weeks of gestation, weighing 2,974 g, via normal vaginal delivery. There was no history of complications during pregnancy or delivery. There was also no significant family history of ophthalmic disease. The right eye had a clear cornea with no abnormalities (Figure 1).

**Figure 2:**
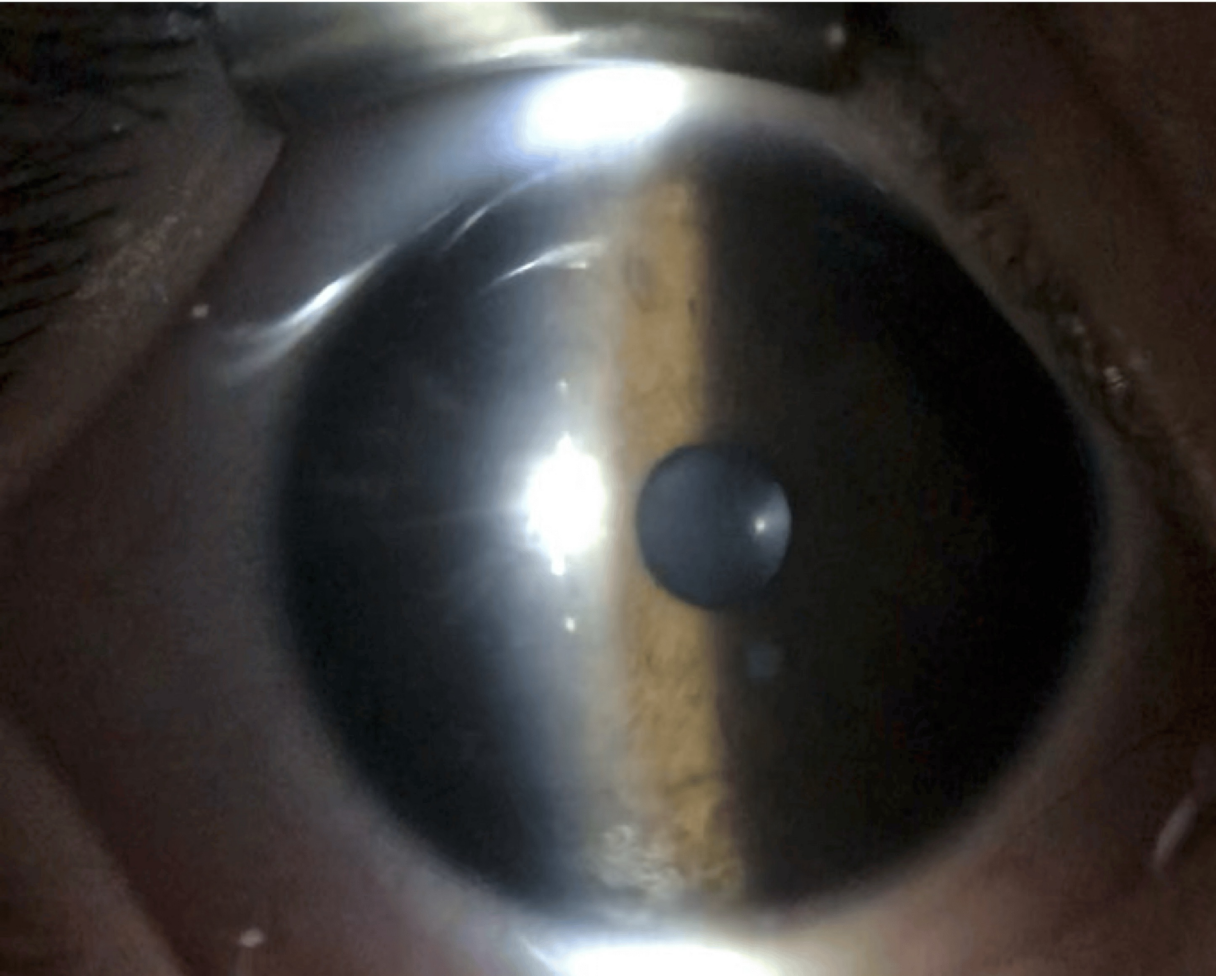
Anterior segment slit-lamp photograph of the right eye showing no significant abnormalities The anterior chamber is deep

The corneal diameter was 11.0 mm in both the vertical and horizontal axes. The depth of the anterior chamber was normal (Figure 2).

**Figure 3:**
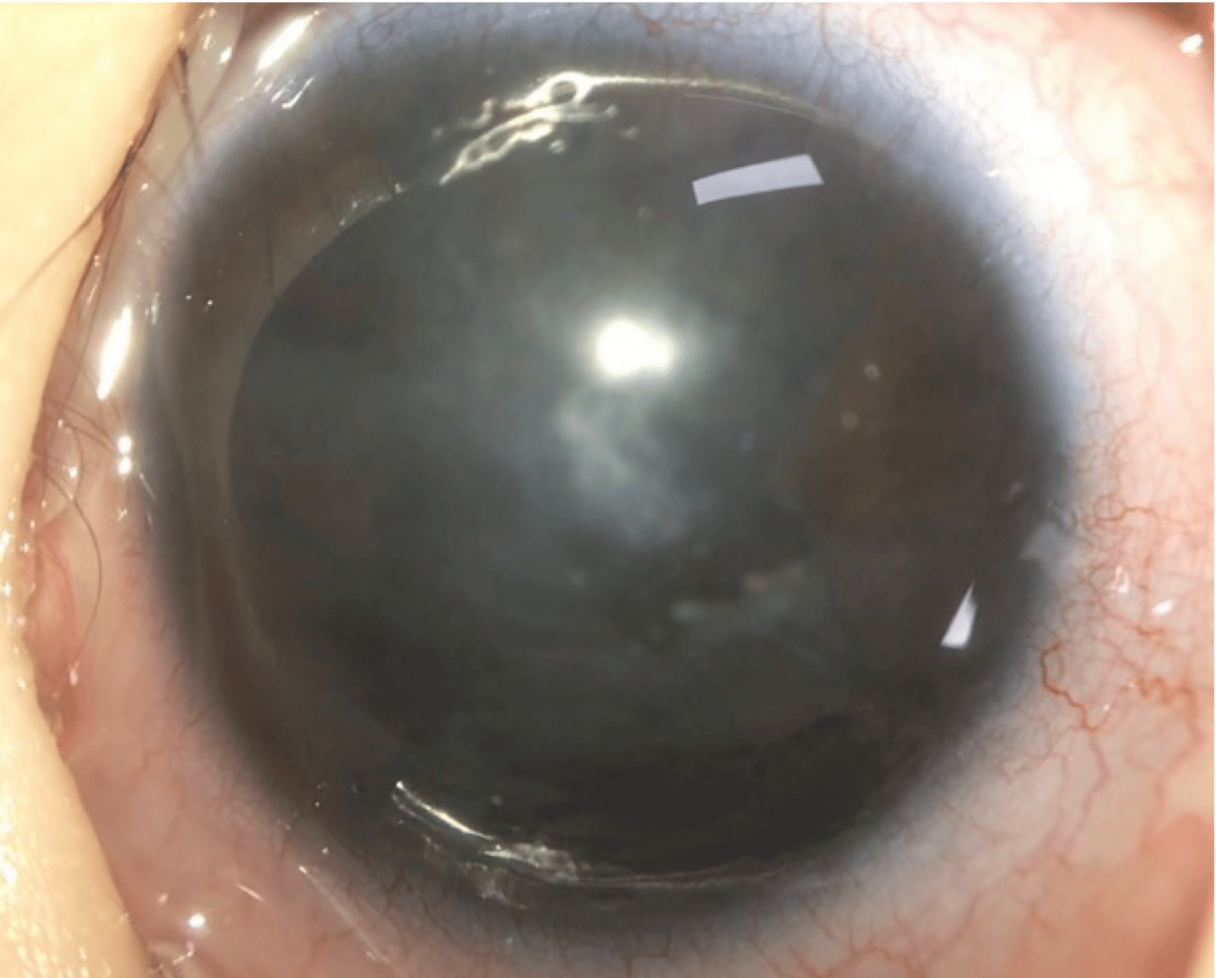
Anterior segment photograph of the left eye Corneal opacity is observed, and the pupil is not clearly visible

The IOP, measured using iCare tonometry (TA01, Icare Finland, Finland), was 10 mmHg. The mean axial length of the right eye was 20.3 mm. In the left eye, the corneal diameter measured 13.0 mm vertically and 13.0 mm horizontally. Corneal opacities were also observed (Figure 3).

**Figure 4:**
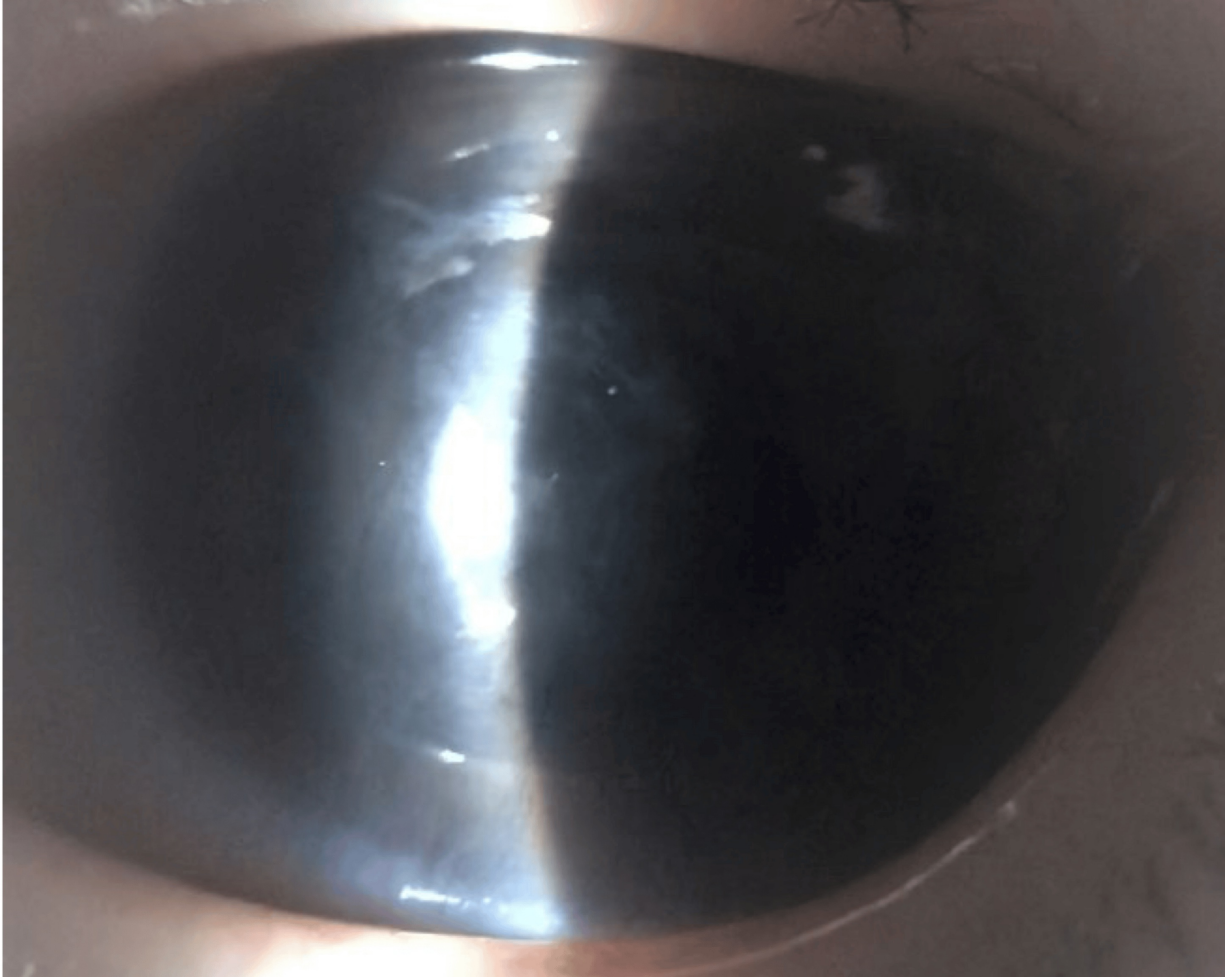
Anterior segment slit-lamp photograph of the left eye The cornea is in contact with the anterior surface of the iris, and the anterior chamber is completely obliterated

The anterior chamber was absent, and the pupil was completely occluded (Figure 4).

**Figure 5:**
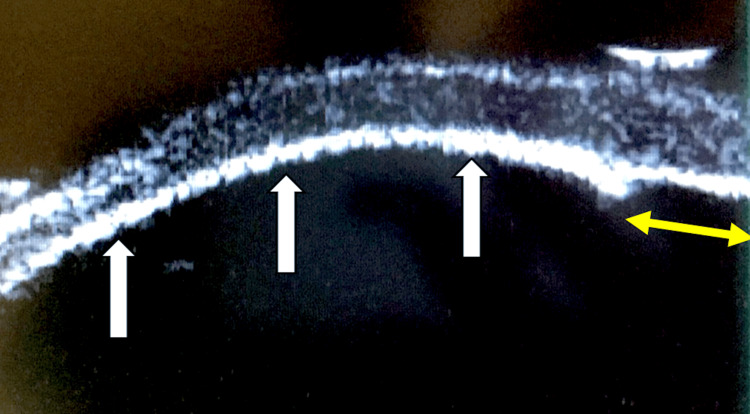
Ultrasound biomicroscopic image of the left eye The posterior surface of the cornea is in contact with the anterior surface of the iris, indicating complete obliteration of the anterior chamber. The three white arrows indicate the iris. A localized thinning area is observed, suggesting the possibility of an incomplete or rudimentary pupil. The yellow double-headed arrow indicates the location of the rudimentary pupil

**Figure 6:**
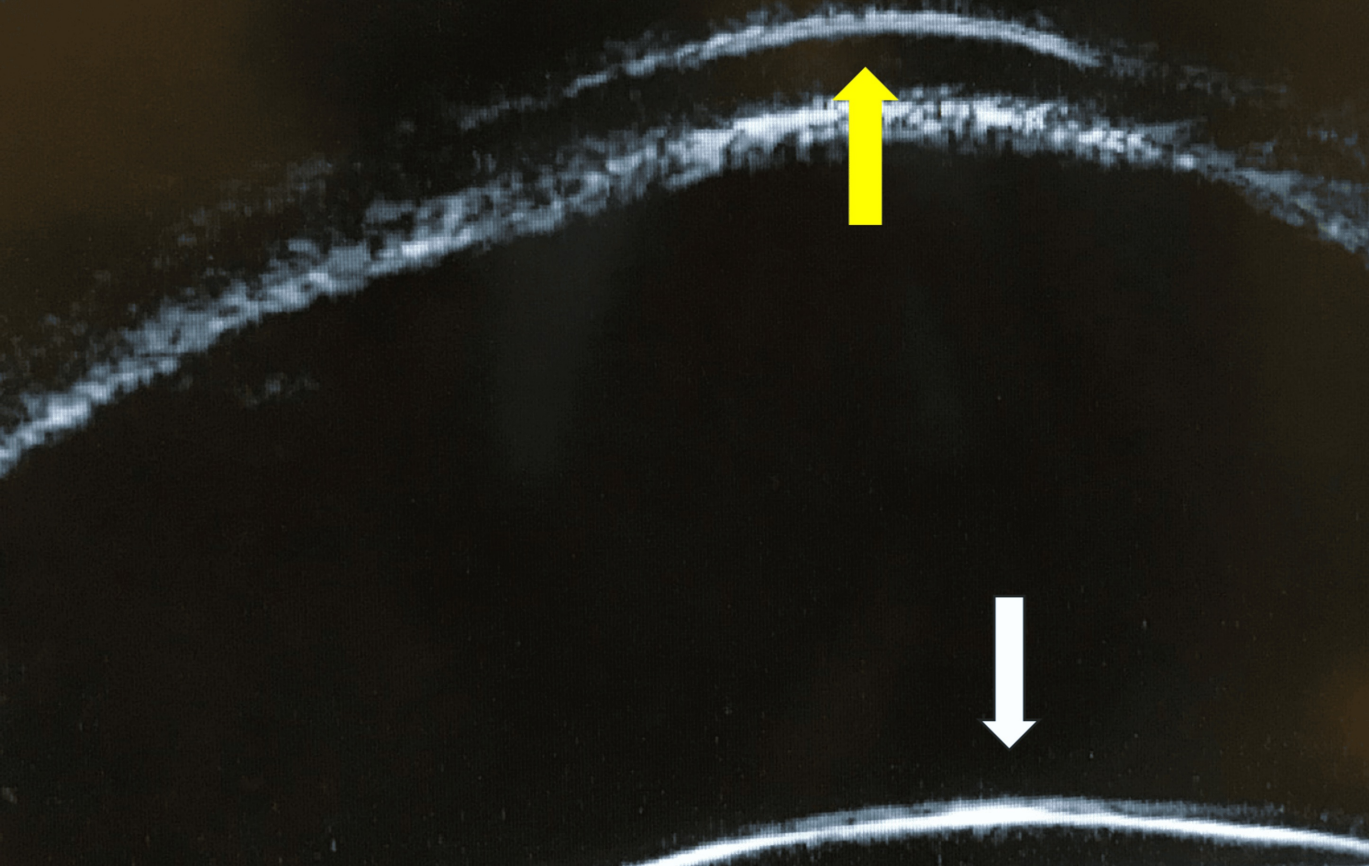
Ultrasound biomicroscopic image of the left eye The crystalline lens is visualized, and no iris tissue is seen between the cornea and lens, suggesting that the structure adherent to the posterior corneal surface is the iris. The white arrow indicates the crystalline lens, and the yellow arrow indicates the cornea

The IOP, measured using iCare tonometry, was 26 mmHg. The axial length was 25.3 mm. B-mode ultrasonography revealed no significant abnormalities. Ultrasound biomicroscopy images were obtained (Figures 5, 6).

**Figure 7:**
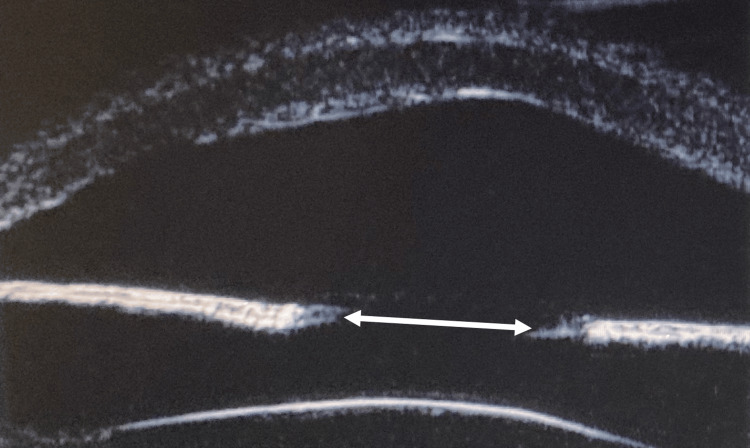
Postoperative ultrasound biomicroscopy image The anterior chamber is reformed with a clearly identifiable pupil and a crystalline lens. The iris appeared thin. The white double-headed arrow indicates the formed pupil

The fundus was not visible due to the presence of acorea. No mydriasis was observed even after instillation of 0.5% tropicamide and 0.5% phenylephrine hydrochloride. The patient was diagnosed with secondary angle-closure glaucoma of the left eye due to acorea. To alleviate angle closure, a pupil was created as an initial step. Under general anesthesia, blunt dissection between the cornea and iris was performed using a viscoelastic substance to secure adequate space for the insertion of a vitreous cutter. The cutter was then introduced through a corneal incision, and central iridectomy was performed to create the pupil. The anterior chamber depth was normalized immediately after pupil formation (Figure 7). Despite pupilloplasty, corneal opacity persisted, and the pupil remained small, which made fundus examination challenging.

The patient exhibited persistently elevated IOP in the left eye despite resolution of the angle-closure mechanism and initial medical management. At one year of age, two months after pupil reconstruction, a 120° trabeculotomy was performed in the superior temporal quadrant, resulting in a transient reduction in IOP. However, IOP subsequently increased, and a second 120° trabeculotomy was performed in the inferior temporal quadrant at one year and one month of age.

Despite these procedures, the IOP remained elevated, peaking above 40 mmHg. At one year and three months of age, an AGV was implanted in the superotemporal quadrant, and the tube was covered with preserved sclera, which led to a substantial but temporary decrease in IOP. Due to the recurrence of elevated IOP, a BGI was subsequently implanted in the superonasal quadrant, and the tube was covered with preserved sclera at one year and six months of age. Following BGI implantation, the IOP was effectively controlled and remained within a normal range.

Throughout the clinical course, the patient was treated intermittently with topical antiglaucoma medications, including a fixed combination of 0.005% latanoprost, 2% carteolol eye drops, and 0.4% ripasudil hydrochloride hydrate, initiated after the first and second trabeculotomies, respectively. The left IOP remained stable between 8 and 12 mmHg, indicating good postoperative control. However, at one year and 11 months of age (six months after BGI implantation), the patient developed swelling of the left eyelid, conjunctival infection, corneal opacity, and vitreous opacity on B-scan ultrasonography. These findings raised a suspicion of endophthalmitis, and the patient was subsequently hospitalized for further management.

Five days before the onset, the patient developed a conjunctival injection in the left eye. Three days prior, the patient had a fever of 38°C and was examined at a local pediatric clinic, where a diagnosis of acute upper respiratory tract infection was made. On the day of onset, the patient developed corneal opacities and marked eyelid swelling in the left eye.

**Figure 8:**
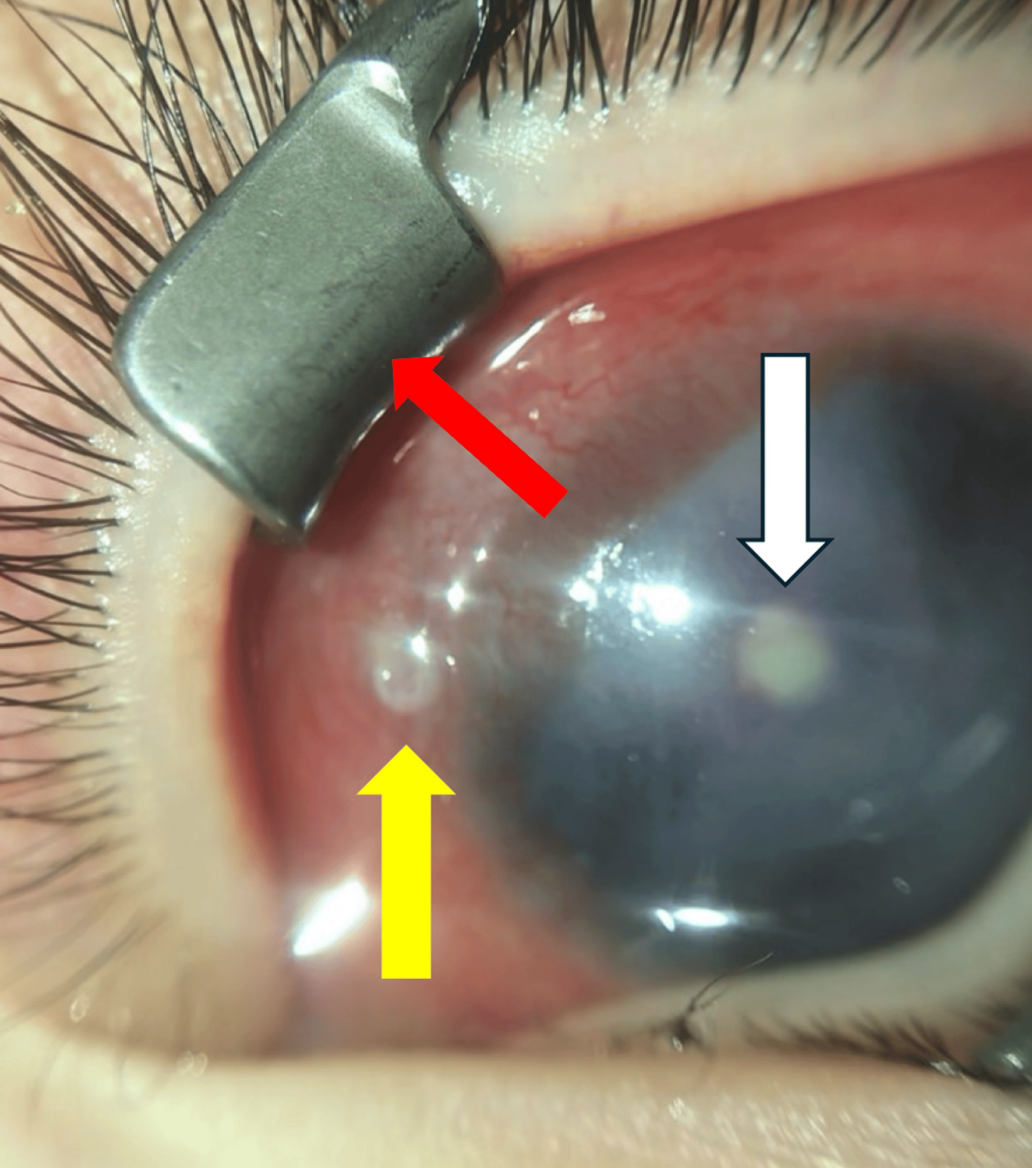
A preoperative photograph of the anterior segment of the left eye Marked ciliary injections and corneal opacities are also observed. White deposits are observed in the corneal endothelium. The white arrow indicates white deposits. An ulcerative lesion is present in the conjunctiva near the limbus at the 11 o'clock position. The yellow arrow indicates a conjunctival ulcer. The red arrow indicates the 12 o’clock direction

The patient presented with a body temperature of 38°C. IOP was 15 mmHg in the right eye and 25 mmHg in the left eye. The left eye exhibited marked eyelid swelling, discharge, conjunctival and ciliary injections, and corneal opacities. Preoperative anterior segment photography of the left eye is shown in Figure 8.

Marked ciliary injections and corneal opacities were observed, along with white deposits on the corneal endothelium. An ulcerative lesion was present in the conjunctiva near the limbus at the 11 o'clock position. No apparent hypopyon was observed in the anterior chamber, and there was no obvious tube exposure. B-scan ultrasonography revealed vitreous opacities, but fundus visualization was not possible. No abnormalities were observed in the right eye.

Laboratory testing revealed an elevated white blood cell count of 15,200/μL (neutrophils, 74%) and a C-reactive protein level of 10.25 mg/dL, indicating a systemic inflammatory response. Ocular discharge was collected for culturing on the day of admission. Empirical topical antimicrobial therapy was initiated with 0.3% gatifloxacin, 0.5% cefmenoxime hydrochloride, and 0.3% tobramycin ophthalmic solutions, each administered hourly. In addition, mydriatic eye drops and 0.3 % ofloxacin ophthalmic ointment were applied.

**Figure 9:**
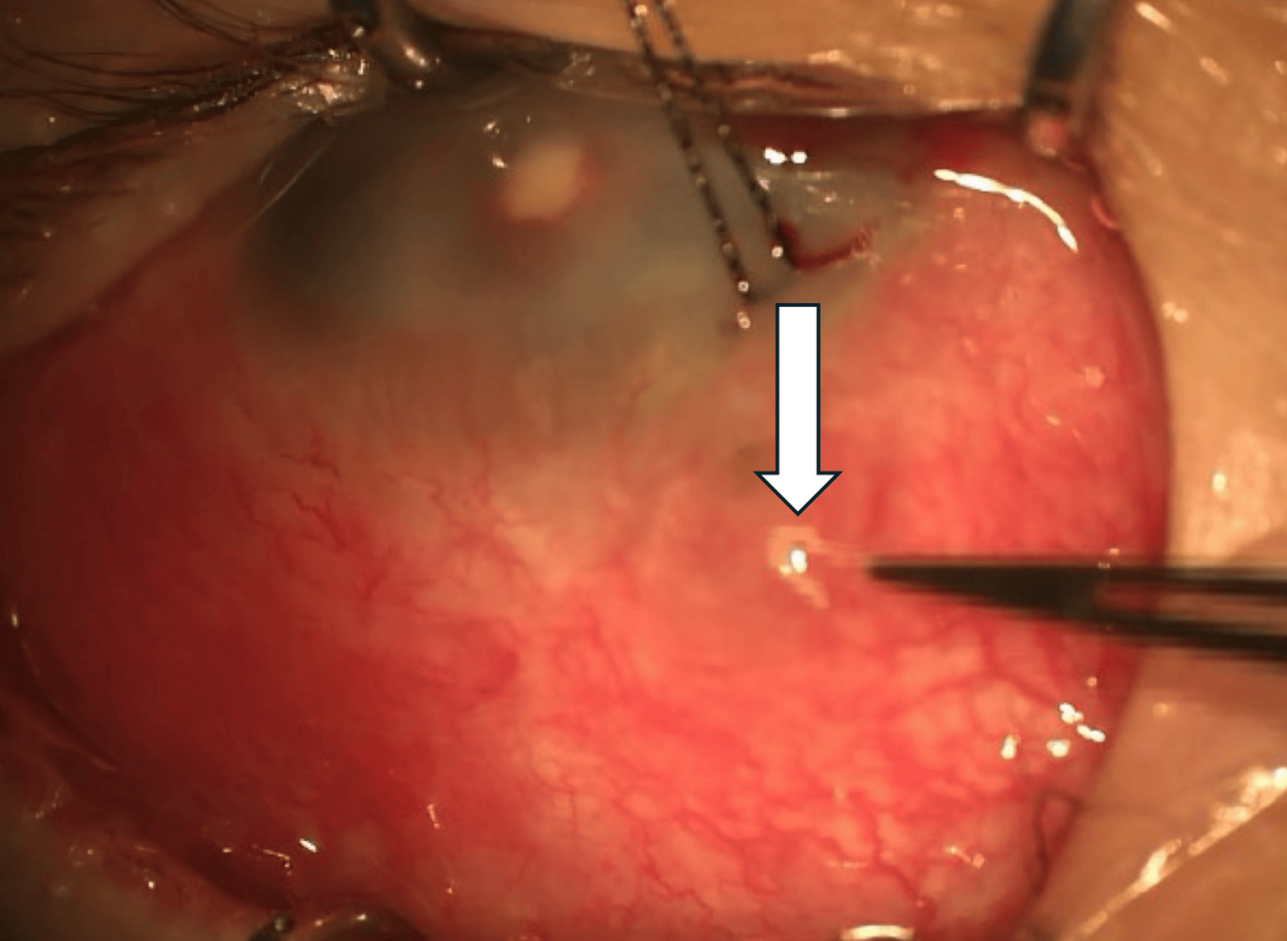
An intraoperative photograph of the anterior segment of the left eye A retained suture, presumably from the previous surgery, is observed at the site of the conjunctival ulcer. The white arrow indicates a retained suture

The following day, blood cultures were obtained, and intravenous antibiotic therapy was initiated with vancomycin hydrochloride (80 mg/kg/day) and cefozopran hydrochloride (100 mg/kg/day), a fourth-generation cephalosporin, in accordance with pediatric infectious disease guidelines and after consultation with specialists. Blood culture results were negative. Although eyelid swelling showed gradual improvement, anterior segment inflammation remained largely unchanged. Therefore, on hospital day 9, anterior chamber irrigation and pars plana vitrectomy were performed on the left eye. At one year and 11 months of age, the patient underwent anterior chamber irrigation, lensectomy, and anterior vitrectomy of the left eye. Intraoperatively, inflammatory adhesions and residual suture material (9-0 silk) were observed at the corneal incision site near the 11 o’clock position (Figure 9).

These were carefully identified and excised, and the suture material was subjected to microbiological culture. No evidence of tube exposure was observed in the conjunctiva at the 1 or 11 o’clock positions, and both the AGV and BGI appeared stable and intact.

Dense white inflammatory deposits adhered to the posterior surface of the cornea and were removed using Simcoe irrigation/aspiration and Ikeda forceps (M.E. Technica, Tokyo, Japan). Anterior chamber fluid was collected for culture. The crystalline lens, iris, and anterior vitreous humor were necrotic and fused into a dense amorphous mass. Tissues were excised using a vitrectomy cutter and irrigation/aspiration. The vitreous fluid was subjected to microbiological analysis. At the conclusion of surgery, intravitreal injections of vancomycin hydrochloride (10 mg/mL, 0.1 mL) and ceftazidime hydrate (20 mg/mL, 0.1 mL) were administered. Subconjunctival injections of vancomycin hydrochloride (10 mg/mL, 0.1 mL), ceftazidime hydrate (20 mg/mL, 0.1 mL), and betamethasone sodium phosphate were administered. Microbiological culture of the anterior chamber fluid and vitreous sample revealed *Haemophilus influenzae* (minimum inhibitory concentrations: cefotaxime >2 µg/mL, nonsusceptible, cefepime 2 µg/mL, susceptible, which was used as a surrogate for cefozopran susceptibility in clinical decision-making), leading to the diagnosis of infectious endophthalmitis.

**Figure 10:**
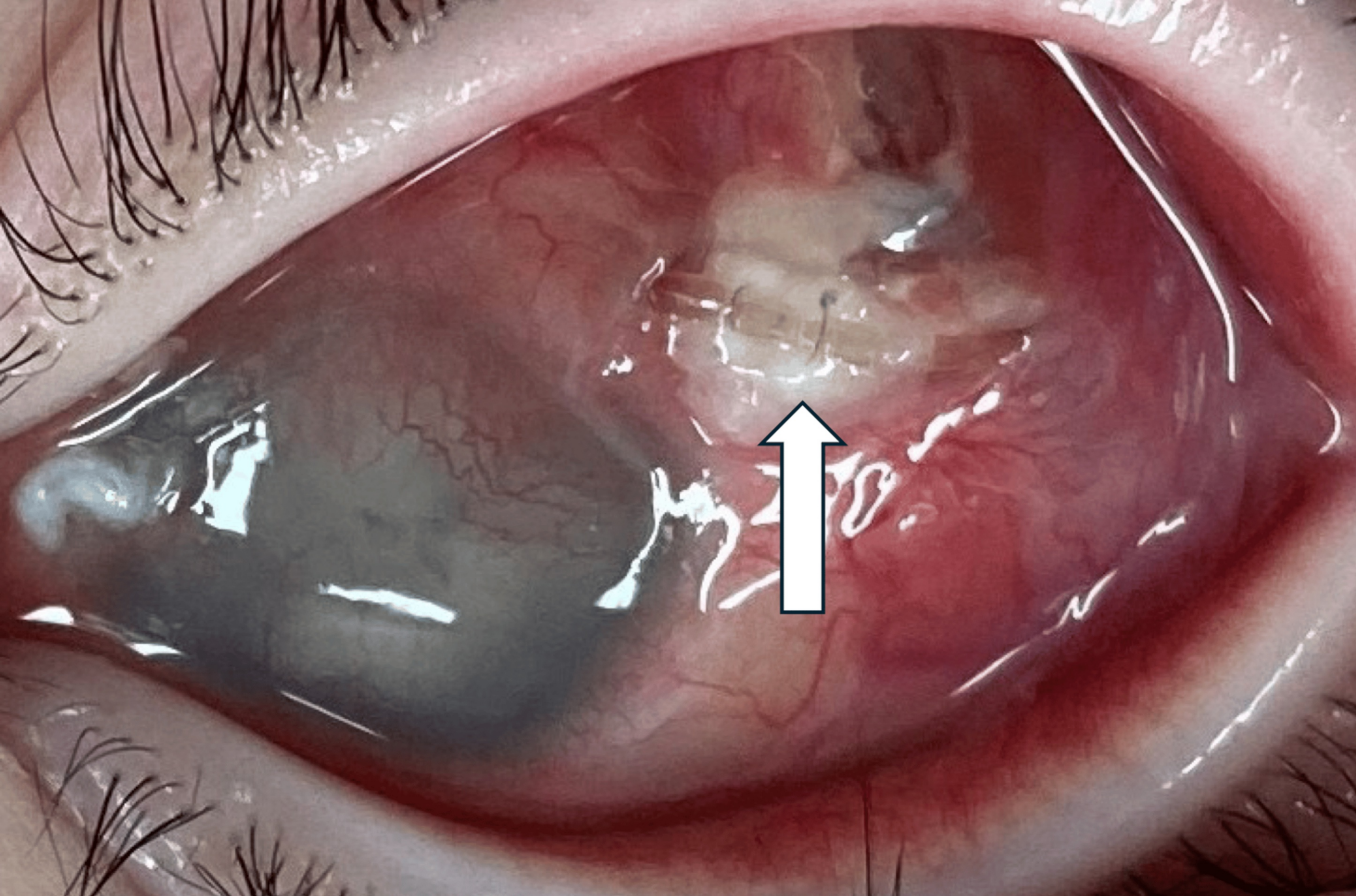
Tube exposure of the Ahmed glaucoma valve is observed Tube exposure of the Ahmed glaucoma valve is observed, and both the Ahmed glaucoma valve and the Baerveldt glaucoma implant are removed. The white arrow indicates the exposed tube

Postoperatively, the patient demonstrated partial improvement in eyelid swelling and spontaneously opened the affected eye. Although corneal opacity and conjunctival hyperemia persisted, no distinct abnormalities, such as membrane formation, were observed in the anterior chamber. The vitreous opacities improved, and no retinal detachment was noted on B-scan ultrasonography. The patient was discharged with continued administration of the following topical medications: 1.0% vancomycin hydrochloride, 0.5% ceftazidime hydrate, and 0.1% betamethasone sodium phosphate ophthalmic solutions. Three months later, exposure of the AGV tubes was observed, and surgical removal of both AGV and BGI was performed (Figure 10). At the time of AGV removal, proliferative vitreoretinopathy had already developed. We decided to remove the BGI as well, because leaving it in place could have led to progression to phthisis bulbi or recurrence of endophthalmitis due to tube exposure. There was strong adhesion; however, the implants could be removed without particular difficulty. Conjunctival coverage was also achieved without problems.

Since then, the patient has not exhibited a recurrence of eyelid swelling or other signs of inflammation; however, the patient’s left eye progressed to phthisis bulbi.

## Discussion

We report a case of unilateral secondary angle-closure glaucoma associated with congenital acorea in a boy who developed endophthalmitis after glaucoma drainage implant surgery. Regarding acorea, reports include two familial cases [1,2] and two cases isolated in Asia [3,4]. In the three pedigree families of the Chinese family, three individuals were affected, whereas four were unaffected. In the four pedigree families of the Japanese family, five individuals were affected, whereas eight were unaffected. Pedigree analysis indicated autosomal-dominant inheritance in this family. However, sporadic cases have also been reported, and the present case, with no notable family history, was considered sporadic.

In the present case, marked secondary angle-closure glaucoma developed in association with the acorea. Al Otaibi et al. and Kandori et al. reported cases of secondary angle-closure glaucoma caused by the pupillary iris-lens membrane [6,7]. There has been one previous report of angle-closure glaucoma associated with acorea. If the pupil is truly absent, the aqueous humor produced by the ciliary body would not be able to pass into the anterior chamber; theoretically, all such cases should develop angle-closure glaucoma. It is presumed that, in most reported cases, microcoria are present, allowing communication between the posterior and anterior chambers. Although angle-closure glaucoma developed in this patient at this age, the possibility of a small pupil could not be completely ruled out.

Treatment options for angle-closure glaucoma include lens extraction and peripheral iridectomy, both of which aim to relieve the pupillary block. However, in cases of acorea, the absence of a pupil disrupts the flow of the aqueous humor between the posterior and anterior chambers. Moreover, because acorea lacks a visual axis, reconstruction of the visual axis is also an important therapeutic goal. In angle-closure glaucoma associated with acorea, surgical creation of the pupil facilitates the formation of the anterior chamber and simultaneously establishes a visual axis, making it a potentially desirable treatment approach. Therefore, pupilloplasty is considered the most suitable surgical procedure.

Several methods for pupilloplasty for treatment of acorea or cases of severe fibrous pupillary membrane have been reported, including scissors [8], forceps [6,8], Ocutome [9], laser [10], radiofrequency diathermy [11], and a vitrector [4,6]. A 25-gauge vitrector was used in this study. Vitrectors are readily available in facilities where vitreous surgery is routinely performed, and many surgeons are familiar with their use. Although the surgeon was not a vitreoretinal specialist, pupilloplasty was readily performed. Therefore, vitrectors may be suitable for pupilloplasty.

Trabeculotomy is the primary surgical option for pediatric glaucoma [12]. Nonetheless, in cases refractory to this intervention, implantation of a glaucoma drainage implant may be required. In this case, although the IOP was controlled following BGI insertion, the patient subsequently developed endophthalmitis. Several studies have investigated the incidence of endophthalmitis following glaucoma drainage implant surgery in children. A retrospective study reported that four of 69 eyes (5.8%) of 52 pediatric patients developed endophthalmitis, necessitating implant removal [13]. Al-Torbak et al. reported a higher incidence of endophthalmitis in children (4.4%, five cases among 113 eyes of 102 patients) compared to adults (0.9%, four cases among 429 eyes of 403 patients) [14]. Other studies have documented an incidence of endophthalmitis after glaucoma drain implantation, ranging from 1.9% to 11.7% [15-18]. Pediatric patients undergoing glaucoma device implantation are at an increased risk of developing endophthalmitis. Therefore, the emergence of clinical signs, such as ocular discharge, conjunctival hyperemia, and ocular pain, should prompt a high index of suspicion for endophthalmitis.

Microbiological cultures revealed *H. influenzae*, a common causative organism of pediatric postglaucoma drainage implant endophthalmitis [14,19,20]. Notably, the patient was diagnosed with an acute upper respiratory tract infection at a local pediatric clinic several days before the onset. Given the concordance between the clinical course and microbial findings, it is plausible that a nasolacrimal transmission related to the preceding upper respiratory tract infection may have served as a trigger for the intraocular infection in this case.

## Conclusions

In conclusion, this case highlights the potential effectiveness of pupilloplasty in managing acorea-associated secondary angle-closure glaucoma. By creating a functional pupil, this procedure not only restores aqueous humor flow and deepens the anterior chamber but also establishes a visual axis, offering both anatomical and functional benefits. At the same time, given the increased susceptibility of children to endophthalmitis after glaucoma drainage implant surgery, clinicians should maintain a high level of vigilance for signs of intraocular infection. Timely intervention can help preserve remaining ocular structures, prevent further complications, and optimize long-term outcomes.
